# Case Report: Simultaneous presentation of end-stage heart failure with cardiogenic shock requiring emergency transplantation in monozygotic twins with myofibrillar myopathy: a previously unknown genetic disease?

**DOI:** 10.3389/fcvm.2025.1673907

**Published:** 2025-12-02

**Authors:** Giovanni Battista Luciani, Cristina Panico, Antonella Galeone, Vittoria Medeghini, Matteo Ciuffreda, Rossana Mineri, Livio San Biagio, Paola Tonin, Gaetano Nicola Vattemi, Stiljan Hoxha, Alessandra Francica, Gina Mazzeo, Alessia Gambaro, Leonardo Gottin, Giuseppe Faggian, Francesco Onorati, Elisa Di Pasquale

**Affiliations:** 1 Division of Cardiac Surgery, Department of Surgery, Dentistry, Pediatrics and Gynecology, University of Verona, Verona, Italy; 2 Cardiovascular Department, IRCCS Humanitas Research Hospital, Milan, Italy; 3 Department of Biomedical Sciences, Humanitas University, Milan, Italy; 4 Department of Biomedical Sciences, Institute for Genetic and Biomedical Research, UOS of Milan, National Research Council of Italy, Milan, Italy; 5 Division of Cardiology, Azienda Ospedaliera Universitaria Integrata di Verona, Verona, Italy; 6 Unit of Medical Genetics, IRCCS Humanitas Research Hospital, Milan, Italy; 7 Division of Cardiac Surgery, Azienda Ospedaliera Universitaria Integrata di Verona, Verona, Italy; 8 Division of Neurology, Azienda Ospedaliera Universitaria Integrata di Verona, Verona, Italy; 9 Division of Neurology, Department of Neurosciences, Biomedicine and Movement Sciences, University of Verona, Verona, Italy; 10 Division of Anesthesiology, Department of Surgery, Dentistry, Pediatrics and Gynecology, University of Verona, Verona, Italy

**Keywords:** heart transplantation (HT), heart failure, cardiomyopathy, genetic testing, myofibrillar myopathy

## Abstract

We report the rare case of 16-year-old male monozygotic twins, born to first cousins, who developed acute end-stage heart failure due to dilated cardiomyopathy, requiring emergency heart transplantations within days of each other. Both twins presented with rapid-onset symptoms and progressed to refractory cardiogenic shock despite inotropic support, necessitating veno-arterial extracorporeal membrane oxygenation. Heart transplantation was performed within 3 weeks of admission, and both twins have remained clinically stable 3 years posttransplantation. Family history was negative for cardiomyopathy. Genetic testing identified the following four shared variants: a homozygous *FLNC* variant (p.Tyr786Asp), two heterozygous *SCN5A* variants (p.Gln1366His and p.Thr1367Ser), and a heterozygous *MYH7* variant (p.Ile1927Phe). All were classified as variants of uncertain significance. Segregation analysis showed that both parents were heterozygous carriers of the *FLNC* variant; their daughter (the twins' sister) was homozygous for *FLNC* and carried both *SCN5A* variants; however, she was asymptomatic. A muscle biopsy from one twin showed pathological features consistent with a diagnosis of myofibrillar myopathy, including fiber disarray and desmin accumulation. *In silico* analysis suggested structural disruption associated with the *MYH7* variant. This case likely represents an unclassified genetic cardiomyopathy with skeletal and cardiac muscle involvement. It underscores the diagnostic complexity of consanguineous pedigrees and the limitations of current variant classification systems. The synchronous disease onset in these monozygotic twins suggests a potential genetic or epigenetic trigger and highlights the value of integrating family studies, histopathology, and computational modeling in the evaluation of inherited cardiomyopathies.

## Introduction

**Figure 1:**
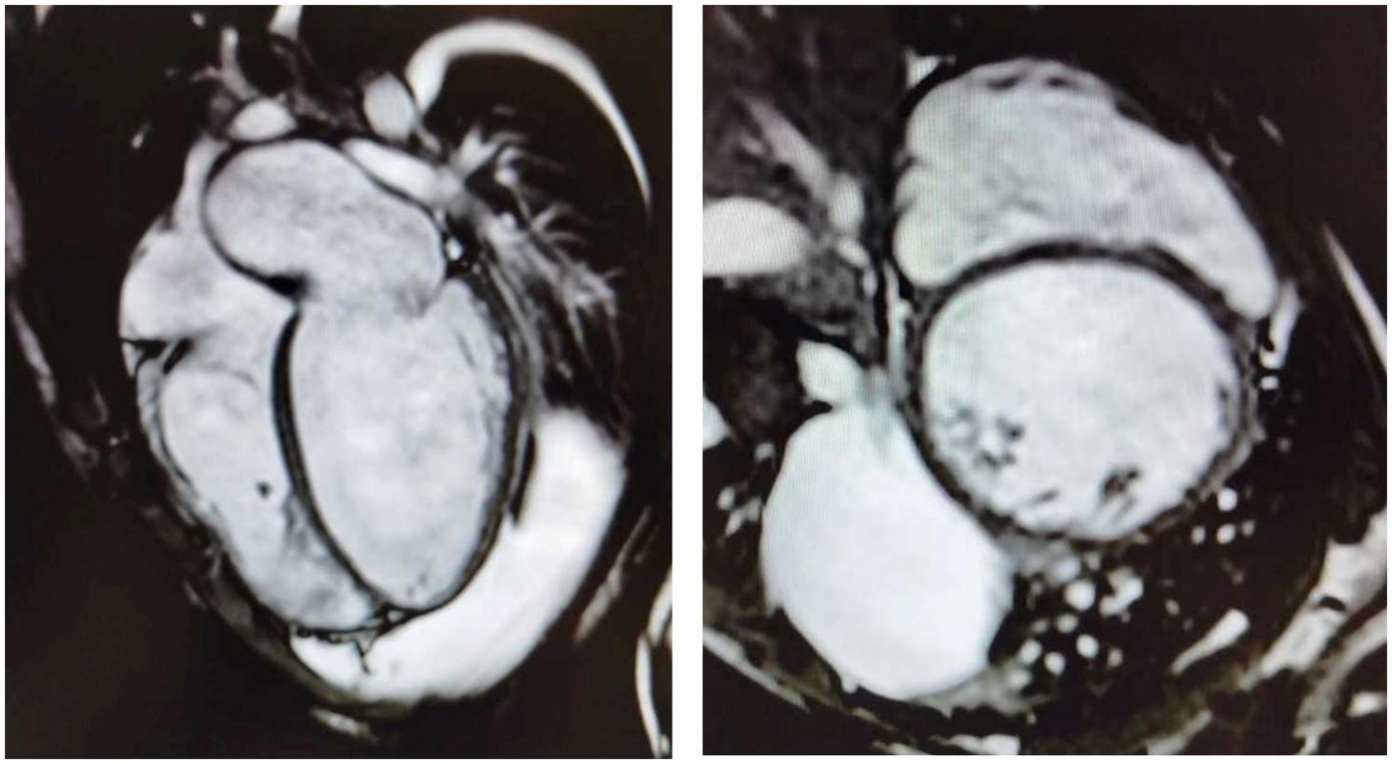
Four-chamber (left) and short-axis (right) cine images from cardiac magnetic resonance imaging demonstrating severe bi-ventricular dilatation, accompanied by myocardial wall thinning and fibrosis.

Herein, we describe the cases of two 16-year-old identical male twins who presented with acute end-stage heart failure (HF) requiring emergency heart transplantation (HT), secondary to dilated cardiomyopathy (DCM). The patients provided informed consent for the publication of their cases. The twins, who are the sons of first cousins, were born at term and were previously in good health, had no past medical history, and no cardiovascular or neuromuscular symptoms. They were diagnosed within 15 days of each other and underwent successful HTs within a week of each other. Twin A had been in good health until he developed a worsening cough and dyspnea. Echocardiography revealed a new diagnosis of DCM with severely reduced systolic function. He was transferred to the coronary care unit (CCU), where intravenous milrinone, furosemide, and norepinephrine were administered. Cardiac MRI confirmed severe biventricular dysfunction with myocardial fibrosis (Figure 1). Myocarditis screening was negative. He was diagnosed with acute HF secondary to idiopathic DCM and listed for an HT (status 2). As his clinical condition worsened to refractory cardiogenic shock, he was transferred to the intensive care unit (ICU), intubated, and placed on femoro-femoral veno-arterial extracorporeal membrane oxygenation (VA-ECMO). Due to progressive left ventricular (LV) distention with pulmonary edema, the patient underwent rescue percutaneous atrial septectomy to obtain LV venting. The patient was listed as status 1 and underwent an HT within 24 days. His postoperative course was complicated by pneumothorax, requiring chest tube insertion. He was discharged on postoperative day 40. He experienced a single episode of acute cellular rejection (grade 2R), which was treated with intravenous methylprednisolone. The patient is thriving 3 years after the HT, with preserved biventricular function and no evidence of cardiac allograft vasculopathy (CAV).

Twin B presented 15 days after his brother, with worsening fatigue and tachycardia. He had been in good health until the appearance of symptoms. Echocardiography showed DCM with severely reduced systolic function. He was transferred to the CCU, where milrinone and norepinephrine were initially administered and later replaced by epinephrine due to severe hypotension. He was diagnosed with familial DCM and listed for an HT. Identical to his twin, his condition rapidly deteriorated to refractory cardiogenic shock, necessitating VA-ECMO support with subsequent LV venting via percutaneous atrial septectomy. He underwent HT 17 days after admission, 1 week after his brother. His postoperative course was complicated by a lower airway obstruction that led to a brief episode of cardiac arrest, after which he was successfully resuscitated without neurological sequelae. He also developed bilateral pneumothorax. He was discharged on postoperative day 50. Similar to his identical twin, this patient is thriving 3 years after transplantation, with good biventricular function and no evidence of acute rejection or CAV.

Family members, including the patients’ parents and a 21-year-old sister, were asymptomatic from a cardiovascular and neuromuscular point of view. Physical examinations, ECGs, and echocardiography of their parents and sister were negative. Their sister is currently asymptomatic and continues active surveillance with yearly clinical examinations, ECG, and echocardiography, while reproductive counseling has not yet been planned.

## Genetic testing

Genetic testing was performed, and the following four variants in three different genes were identified in both twins:

1. Homozygous c.2356T > G in the *FLNC* gene (NM_001458), resulting in p.Tyr786Asp.
2. Heterozygous c.4098G > T in the *SCN5A* gene (NM_198056), resulting in p.Gln1366His.
3. Heterozygous c.4099A > T in the *SCN5A* gene (NM_198056), resulting in p.Thr1367Ser.
4. Heterozygous c.5779A > T in the *MYH7* gene (NM_000257), resulting in p.Ile1927Phe.

**Figure 2:**
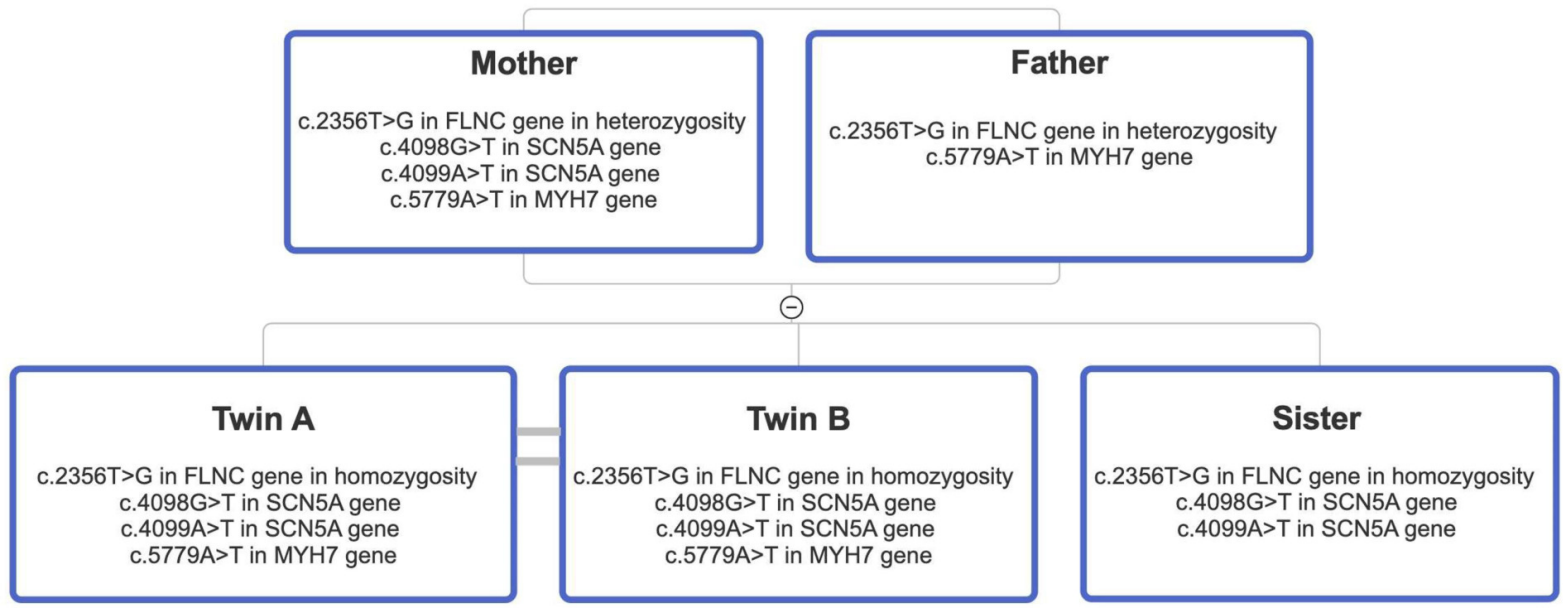
Pedigree diagram illustrating the segregation analysis performed in the family of the two probands. Notably, both parents are heterozygous carriers of both the *MYH7* and *FLNC* variants. In contrast, the sister is homozygous for the *FLNC* variant and wild-type for the *MYH7* variant.

**Figure 3:**
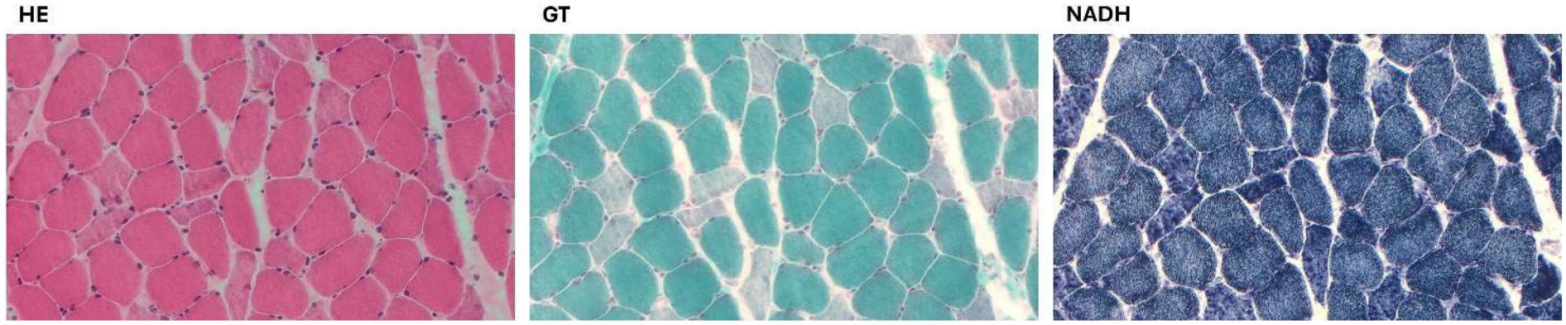
A biopsy of the deltoid muscle showing increased fiber size variation, rimmed vacuoles in a few fibers, and several fibers with amorphous eosinophilic material, loss/reduction of ATPase activity, and an irregular intermyofibrillar network. (HE, hematoxylin and eosin; GT, modified Gomori trichrome; NADH, nicotinamide adenine dinucleotide-tetrazolium reductase).

**Figure 4:**
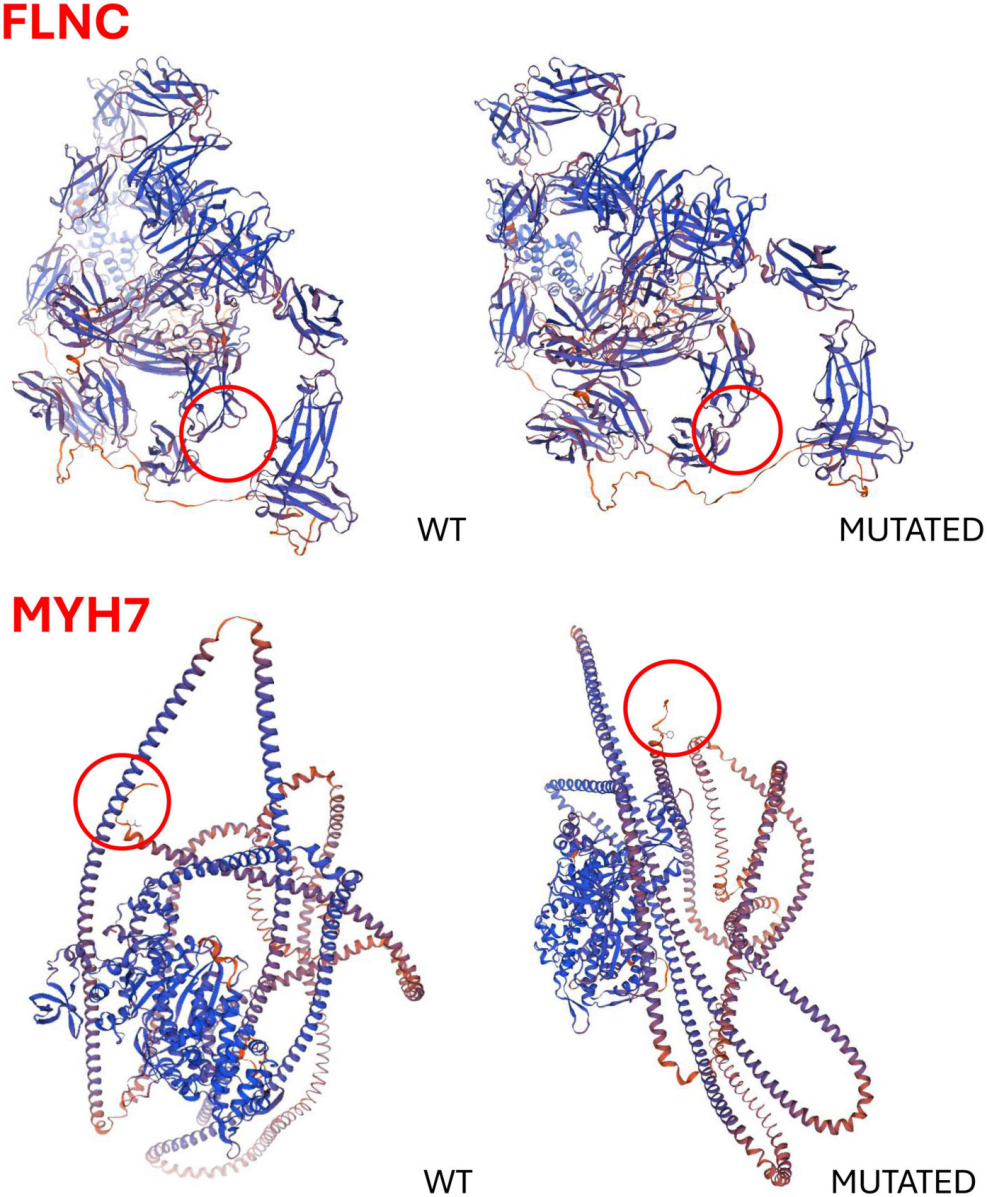
Predicted three-dimensional structures of the wild-type and mutant FLNC and MYH7 proteins. *In silico* analyses were performed using SWISS-MODEL and AlphaFold to assess the structural impact of the identified variants. Notably, only the *MYH7* variant appears to significantly alter the protein's three-dimensional conformation.

The *SCN5A* variants have not been previously described, and their significance remains undetermined. The *MYH7* variant has been linked to hypertrophic cardiomyopathy, but its role in DCM remains unclear. The *FLNC* variant, absent from control databases, is classified as of uncertain clinical significance according to the American College of Medical Genetics and Genomics (ACMG) criteria. A family segregation study was conducted (Figure 2). The mother presented with the *FLNC* variant in heterozigosity together with both *SCN5A* variants and the *MYH7* one. The father was found to carry the heterozygous *FLNC* and *MYH7* variants, while the sister was found to be a carrier of the *FLNC* variant in homozygosity, along with both *SCN5A* variants. Clinical examinations and echocardiography of the family members revealed no cardiovascular disease. A biopsy of the deltoid muscle was performed on one of the two siblings during the index hospitalization, which showed increased fiber size variation, rimmed vacuoles, loss/reduction of ATPase activity, and an irregular intermyofibrillar network (Figure 3). Using immunofluorescence, irregular areas of increased reactivity for desmin, αB-crystallin, and myotilin were observed within the abnormal fibers. The histological and immunohistochemical findings were suggestive of myofibrillar myopathy (MFM). Given the suspicion of a pathogenic role of the *FLNC* and *MYH7* variants, an *in silico* analysis was performed, and the wild-type and mutant protein structures were predicted using SWISS-MODEL and AlphaFold. Only the *MYH7* gene variant appeared to the associated to an altered three-dimensional structure of the protein (Figure 4).

## Discussion

DCM is characterized by ventricular dilation and systolic dysfunction, often leading to HF requiring an HT (1). Genetic factors play a central role in the pathogenesis of DCM, with mutations in genes encoding cardiac sarcomeric proteins, cytoskeletal components, and other critical proteins. The key genes implicated in inherited forms of DCM include *TTN*, *LMNA*, *SCN5A*, *MYH7*, and *FLNC*, among others (2). Variants of uncertain significance (VUSs) are often identified in genetic testing, which complicates diagnosis and clinical management (3). In the case of these twins, we report a previously unreported form of genetic cardiomyopathy, where the identification of VUSs in three genes, namely, *MYH7*, *SCN5A*, and *FLNC*, posed challenges in determining the pathogenic role of these variants. Family segregation studies help clinicians to identify whether a variant segregates with disease, offering an insight into its pathogenicity. However, the utility of segregation studies is limited in consanguineous families. Indeed, close genetic relationships increase the likelihood of shared genetic variants, potentially leading to false-positive associations, in which benign variants appear to cosegregate with disease (4). In this case, the consanguinity of the parents complicated the interpretation of the genetic results. A muscle biopsy played a fundamental role in establishing the final diagnosis of MFM. MFMs are a group of neuromuscular disorders characterized by clinical and genetic heterogeneity (5). The clinical spectrum mainly consists of progressive muscle weakness in the upper and/or lower limbs, which is frequently associated with cardiomyopathy. Causative genes include *DES*, *CRYAB*, *MYOT*, *LDB3*, *FLNC*, *MYH7*, and *BAG3*, which encode for Z-disk or Z-disk-associated proteins, and the disease is usually transmitted as an autosomal dominant trait (6–8). *In silico* analyses can predict the impact of genetic variants on the three-dimensional structure and interactions of proteins (9). In our study, these analyses identified a potentially pathogenic role for the *MYH7* gene variant. Regardless of the molecular aspects of the disease, the exact cause of the abrupt and simultaneous presentation of congestive HF in these identical twins remains elusive. Whether a silent timer of disease onset is concealed in this previously unknown genetic variant or whether psychological/social factors may have elicited the abrupt appearance of cardiogenic shock is presently unclear. At present, we have to assume that this dramatic and timed presentation may be inscribed in the rare genetic mutations associated with this disease.

This clinical case also underscores the fact that genetic diagnosis in cardiomyopathy does not end with variant identification, but also extends to family counseling and follow-up. Integrating genetic testing, clinical surveillance, and reproductive counseling for all relatives, whether symptomatic or not, represents the best medical practice (10) and is an ethical responsibility.

## Conclusion

Since a clear diagnosis of a previously reported genetic cardiomyopathy has not yet been reached in this familial case, we believe this to be a newly identified genetic cardiomyopathy. It is important to note that monozygotic twins offer a unique opportunity to investigate the genetic basis of complex diseases such as DCM. The identification of variants of uncertain significance, the complexity of family segregation studies in consanguineous families, and the complementary role of muscle biopsies in confirming the diagnosis are all important aspects of this case. Genetic testing, alongside clinical evaluation and muscle biopsy, plays a crucial role in improving the accuracy of the diagnosis, while also pointing to the need for further research into the functional role of specific mutations.

## Data Availability

The original contributions presented in the study are included in the article/Supplementary Material, further inquiries can be directed to the corresponding author/s.
